# Standardized nursing terminology for adult emergency services: mapping and validity

**DOI:** 10.1590/0034-7167-2024-0506

**Published:** 2026-01-26

**Authors:** Tatiane Félix Barbosa de Queiroz, Priscilla Alfradique de Souza, Natália Chantal Magalhães da Silva, Rosane Barreto Cardoso, Rodrigo Jensen, Daniella Fernandes de Almeida Santos

**Affiliations:** I Universidade Federal do Estado do Rio de Janeiro. Rio de Janeiro, Rio de Janeiro, Brazil; II Universidade Federal do Rio de Janeiro. Rio de Janeiro, Rio de Janeiro, Brazil; III Universidade de São Paulo. São Paulo, São Paulo, Brazil

**Keywords:** Standardized Nursing Terminology, Nursing Process, Emergency Service, Hospital, Adult, Validation Study., Terminología Normalizada de Enfermería, Proceso de Enfermería, Servicio de Urgencia en Hospital, Adulto, Estudio de Validación.

## Abstract

**Objectives:**

to map and validate standardized nursing terminology linkages applied to adult clinical-surgical hospital emergency care.

**Methods:**

this methodological study was conducted in an adult clinical-surgical hospital emergency care unit in Goiânia, Goiás, from July 2022 to January 2023. The study was divided into three stages: collection of signs and symptoms from patient records; cross-mapping by researchers and nurses working in emergency care; and content validity by experts.

**Results:**

twenty-nine terminology linkages were validated, including 16 problem-focused nursing diagnoses, 12 risk diagnoses, and one syndrome diagnosis, as well as 26 nursing outcomes, 96 interventions, and 281 activities. The average agreement was 0.87 across the six Functional Health Standards.

**Conclusions:**

the validated linkages promote the standardization of nursing diagnoses, outcomes, and interventions, increasing the accuracy and efficiency of the nursing process in emergency rooms.

## RESUMEN

**Objetivos:**

mapear y validar la terminología estandarizada de enfermería aplicada a la atención de urgencias hospitalarias clínico-quirúrgicas para adultos.

**Métodos:**

estudio metodológico realizado en una unidad de urgencias hospitalarias clínico-quirúrgicas para adultos en Goiânia, Goiás, entre julio de 2022 y enero de 2023. El estudio se dividió en tres etapas: recopilación de signos y síntomas de las historias clínicas de los pacientes; mapeo cruzado por investigadores y enfermeros que trabajan en urgencias; y validación de contenido por expertos.

**Resultados:**

se validaron veintinueve vínculos terminológicos, incluyendo 16 diagnósticos de enfermería centrados en problemas, 12 diagnósticos de riesgo y un diagnóstico de síndrome, así como 26 resultados de enfermería, 96 intervenciones y 281 actividades. La concordancia media fue de 0,87 entre los seis Estándares de Salud Funcional.

**Conclusiones:**

los vínculos validados promueven la estandarización de los diagnósticos, resultados e intervenciones de enfermería, aumentando la precisión y la eficiencia del proceso de enfermería en los servicios de urgencias.

## INTRODUCTION

Emergency and urgency units (EUU) are gateways for patients with acute conditions and imminent risk of death. At the same time, qualified care is required for individuals seeking these facilities. Therefore, team members must maintain clear communication and understand the workflows to respond dynamically to each patient’s unique needs, given the high turnover and complexity of this setting, which reinforces the need for swift and efficient action^(1,2)^.

Among the professionals working in this scenario, nurses are challenged to provide effective clinical care through agile and personalized assistance, based on solid knowledge, technical skills, and effective care and staff management, aiming at the rapid resolution of patients’ needs^(1)^. Thus, given the computerized resources available in this environment, the electronic medical record, a collection of information on each user, has within its structure the possibility of implementing the Nursing Process (NP), a systematic method composed of five cyclical and interactive stages that guide the recording and execution of the care provided by a nursing team^(3,4)^.

Studies show that NP applicability meets the recommendations of Resolution 736/2024 of the Federal Nursing Council, providing: accurate clinical reasoning and qualification in its actions; improvements in nursing records; organization, quality and safety in care; time and work process optimization; patient and professional satisfaction; contributions with benefits for the institution, patients and employees^(4,5)^.

To ensure the feasibility of this process, the use of standardized nursing terminologies (SNT) and scientific communication with classifications is recommended. Notable among these are NANDA International (NANDA-I) for nursing diagnoses (ND), the Nursing Outcomes Classification (NOC), which measures nursing outcomes (NO), and the Nursing Interventions Classification (NIC), which organizes nursing interventions (NI)^(6-8)^, which, when integrated, are called NNN linkage. Consequently, they are enhanced when healthcare institutions base their practices on institutional philosophy, offering care aligned with patient needs^(6-11)^.

Studies indicate that SNT improve communication among healthcare professionals and optimize decision-making, especially in highly complex settings^(11)^. Records constructed with these terminologies for computerized hospital EUU are considered functional technological tools for organizing, managing, documenting, and demonstrating nursing team care and assistance^(12)^. Furthermore, structuring these terminologies in line with theoretical models enhances systematization of care, contributing to more targeted, effective, and safe care^(9,10)^.

Among the theoretical models that guide data collection and care plan definition, Functional Health Patterns (FHP), developed by Marjory Gordon in 1982, stand out. This typology is structured in 11 categories, including: health perception-health management; nutritional-metabolic; elimination; cognitive-perceptual; self-perception-self-concept; role-relationship; sexuality-reproductive; coping-stress tolerance; value-belief; activity-exercise; and sleep-rest^(9,10)^.

In literature, the validity of instruments for recording NP constructed with SNT guided by a theoretical model provides a description of documentation of emergency nurses’ practice, with legal, technical, ethical, and scientific support^(13)^.

Although studies reveal SNT’s contributions to healthcare, especially in hospital emergencies, which frequently treat unstable patients with a high risk of deterioration, studies that list complete nursing terminologies for this scenario are incipient, characterizing this study as pertinent in view of the elucidated.

## OBJECTIVES

To map and validate Standardized Nursing Terminology connections applied to adult clinical-surgical hospital emergency care.

## METHODS

### Ethical aspects

This study was approved by the *Universidade Federal do Rio de Janeiro* Research Ethics Committees, as the proposing institution, and the *Universidade Federal de Goiás*, as the co-participating institution.

### Study design, period and place

This is a methodological study that used Strengthening the reporting of observational studies in epidemiology^(14)^ as a methodological guide. The study was developed in three stages, from July 2022 to January 2023, in an adult clinical-surgical hospital emergency room of a private high-complexity institution located in Goiânia, Goiás.

The first stage, carried out in July 2022, consisted of collecting signs and symptoms that motivated patients to seek emergency care. The second stage, carried out from August to November 2022, involved cross-mapping construction^(15)^, developed by nurses working in the emergency unit and researchers. The third stage took place from December 2022 to January 2023, with content validity carried out by experts.

### Population or sample; inclusion and exclusion criteria

Medical records were selected through unintentional sampling. A total of 900 medical records containing signs and symptoms recorded during nursing consultations were included, covering visits conducted between January 2019 and December 2021, both daytime and evening.

Fifteen nurses working in the emergency room were invited, with nine participating. Inclusion criteria were having worked in the emergency room on a full-time basis or covering 12 hours of shifts. Exclusion criteria were being a nurse with COVID-19 during the study period.

Forty-seven potential experts were selected by intentional non-probability sampling and the snowball method^(16)^. Inclusion criteria were based on Fehring criteria adaptation^(17)^, requiring a minimum score of 5 points. The scores were distributed as follows: doctoral degree in nursing (4 points); master’s degree in nursing (3 points); doctoral degree and/or master’s degree with studies focused on ND, nursing theory, NP or SNT (2 points); specialization in urgency and/or emergency (2 points); experience in urgency and/or emergency care for a period equal to or greater than 1 year (1 point); publication of articles on NP, FHP, SNT or urgency/emergency (2 points); and participation in scientific events in the last two years related to the study topic (1 point). Specialists who completed the form incompletely were excluded.

### Study protocol

The signs and symptoms extracted from medical records were manually collected by one of the researchers and entered into a Microsoft Excel spreadsheet, where they were organized by FHP and quantified.

Subsequently, nurses working in EUU received a Google Forms link consisting of three sections: an invitation letter, an Informed Consent Form (ICF), and a profile questionnaire (age, sex, length of service, monthly income, and specialization). After collecting this information, videoconferences were held among nurses working in EUU and the researchers to construct cross-mapping^(15)^.

In the form, each section corresponded to an FHP, containing the title with definition, signs and symptoms, NNN linkages, corresponding to NANDA-I 2021-2023^(6)^, NOC 2022^(7)^, and NIC 2022^(8)^ classifications, as well as a space for suggestions and comments. This stage lasted 20 consecutive days. Subsequently, cross-mapping content validity was performed by experts.

Experts received a Google Forms form consisting of four sections: invitation letter; informed consent form; characterization questionnaire (sex, age, place of residence, professional information); and cross-mapping to validate the content of NNN linkages. The linkages were validated on three axes (1) clarity; 2) objectivity; and 3) importance), using a Likert scale composed of five options (A - totally agree (1.0); B - agree (0.75); C - partially (0.5); D - disagree (0.25); E - totally disagree (0.0)^(16)^, with the possibility of selecting one option on each axis. At the end of each section, there was a space for comments and suggestions.

### Analysis of results and statistics

Experts assessed the degree of agreement and reliability of NNN linkages using the Content Validity Index (CVI)^(16,17)^ per set, FHP, and in full. The reference value for reliability and excellence adopted was ≥ 0.80^(16,17)^. To calculate the CVI for each item, the total number of options (5) assigned by experts was considered, divided by the total number of responses.

Microsoft Excel was used to tabulate the data and then perform descriptive analysis. Descriptive statistics were used for the analysis, which consists of a means of organizing and summarizing the main characteristics observed in a data set, allowing the researcher to better understand the data studied^(16)^.

It is noteworthy that the new edition of NANDA-I 2024-2026^(18)^ presents a review of all validated ND. Among them, 17 present modifications in the definition, editing, additions and/or removals of defining characteristics, risk factors and related factors, as well as the replacement of 12 NDs, five of which are risk-related and seven focused on the problem. As for the most current version of NOC 2024^(19)^, there were no modifications in the validated NO.

## RESULTS

A total of 2,267 symptoms were identified, which, after being distributed across eight FHP, guided the cross-mapping of the 76 NNN linkages with 64 different NDs, 59 NOs, and 122 groups of NIs, which resulted in the survey of 602 nursing activities (NA). Chart 1 presents a summary of the established NNN linkages.

**Chart 1:** Functional Health Patterns, signs and symptoms with occurrences, and main terminologies of NANDA-International, Nursing Outcomes Classification and Classification of Nursing Interventions listed for adult clinical-surgical hospital emergencies, Goiânia, Goiás, Brazil, 2023

2^nd^ Functional Health Pattern: Nutritional-metabolic				
***Signs and symptoms grouped by occurrence:*** Gastrointestinal changes, cardiac changes, systemic changes, blood pressure changes, discomfort, fever, systemic changes, inflammatory signs (n=1,189)				
**Nursing diagnoses**		**Nursing outcomes**		**Nursing interventions**
Risk for ineffective thermoregulation		Thermoregulation		Temperature regulation
Impaired spontaneous ventilation		Respiratory status: gas exchange		Respiratory monitoring
**3^rd^ Functional Health Pattern:** Elimination				
***Signs and symptoms grouped by occurrence:*** Diarrhea, diuresis, urinary incontinence (n=367)				
**Nursing diagnoses**		**Nursing outcomes**		**Nursing interventions**
Urinary retention		Urinary elimination		Urinary retention care
Diarrhea		Bowel continence		Diarrhea management
**4^th^ Functional Health Pattern:** Cognitive-perceptual				
***Signs and symptoms grouped by occurrence:*** Trauma, falls, pain, confusion, decreased level of consciousness (n=24)				
**Nursing diagnoses**		**Nursing outcomes**		**Nursing interventions**
Impaired comfort		Comfort status: physical		Environmental management: comfort
Acute pain		Comfort status		Acute pain management
**5^th^ Functional Health Pattern:** Self-perception-self-concept				
***Signs and symptoms grouped by occurrence:*** Speech changes (n=20)				
**Nursing diagnoses**		**Nursing outcomes**		**Nursing interventions**
Impaired verbal communication		Communication		Self-esteem enhancement
**8^th^ Functional Health Pattern:** Coping-stress tolerance				
***Signs and symptoms grouped by occurrence:*** Stress, euphoria, sadness, anxiety, work overload (n=211)				
**Nursing diagnoses**		**Nursing outcomes**		**Nursing interventions**
Fear		Discomfort level		Coping enhancement
**9^th^ Functional Health Pattern:** Value-belief				
***Signs and symptoms grouped by occurrence:*** Sensations and feelings (n=20)				
**Nursing diagnoses**		**Nursing outcomes**		**Nursing interventions**
Spiritual distress		Personal resiliency		Resilience promotion
**10^th^ Functional Health Pattern:** Activity-exercise				
***Signs and symptoms grouped by occurrence:*** Motor disorders and fatigue (n=20)				
**Nursing diagnoses**		**Nursing outcomes**		**Nursing interventions**
Impaired physical mobility		Mobility		Self-care assistance: transfer
**11^th^ Functional Health Pattern:** Sleep-rest				
***Signs and symptoms grouped by occurrence:*** Insomnia (n=13)				
Insomnia		Sleep		Sleep improvement

In relation to FHP representation, the second (nutritional-metabolic) presented 1,189 signs and symptoms, followed by the third (elimination), with 367, and the eighth (coping-stress tolerance), with 211. In contrast, the eleventh (sleep-rest) was the least recurrent, with 13 terms.

Regarding the nine nurses with care practice in EUU participating in the second stage of the study, the predominant age range was 25 to 40 years (44.5%), with the majority being female (67%), 1 to 5 years of experience (55.5%), monthly income between one and three minimum wages, and specialized in urgency, emergency or Intensive Care Unit (44.5%).

As for experts who participated in the content validity stage, the predominant age range was between 30 and 35 years old, with three experts (60%). The majority were male (60%), and four experts (80%) resided in Brazil, while one (20%) was from Colombia. Regarding professional affiliation, most had a long time of teaching experience (60%) and also had practical experience in the emergency room (60%), although none of them had completed a *lato sensu* specialization in EUUs. Scores on the inclusion criteria ranged from 9 to 15, with an average of 11.6.

During content validity by experts, some NOs were suggested for replacement and NAs added, resulting in modifications to three FHPs: nutritional-metabolic; elimination; and perceptive-cognitive. A total of 29 NNN linkages were validated, consisting of 16 problem-focused ND, 12 risk-focused ND, and one syndrome-focused ND, in addition to 26 different NO and 96 NI. The linkages were distributed among six FHPs: 2^nd^ - nutritional-metabolic (0.87); 3^rd^ - elimination (0.84); 4^th^ - perceptive-cognitive (1.0); 8^th^ - coping-stress tolerance (0.90); 9^th^ - value-belief (0.80); and 10^th^ - activity-exercise (0.90). The validated linkages were listed with codes for their respective terminologies to facilitate implementation in practice through patients’ electronic medical record, using software such as that provided in the local emergency room (Chart 2).

**Chart 2:** Functional Health Patterns with the main NANDA-International, Classification of Nursing Outcomes and Classification of Nursing Interventions linkages validated by experts, Goiânia, Goiás, Brazil, 2023

2^nd^ Functional Health Pattern: Nutritional-metabolic						
**Nursing diagnoses**	**Code**	**Nursing outcomes**	**Code**	**Nursing interventions**	**Code**	**CVI**
Risk for impaired skin integrity	00047	Tissue integrity: skin and mucous membranes	1101	Positioning	0840	0.93
Risk for ineffective thermoregulation	00274	Thermoregulation	0800	Temperature regulation	3900	0.86
Impaired spontaneous ventilation	00033	Respiratory status	0415	Airway management	3140	1.0
**3^rd^ Functional Health Pattern:** Elimination						
**Nursing diagnoses**	**Code**	**Nursing outcomes**	**Code**	**Nursing interventions**	**Code**	**CVI**
Diarrhea	00013	Bowel continence	0500	Diarrhea management	0460	0.80
**4^th^ Functional Health Pattern:** Cognitive-perceptual						
**Nursing diagnoses**	**Code**	**Nursing outcomes**	**Code**	**Nursing interventions**	**Code**	**CVI**
Impaired comfort	00214	Comfort status: physical	2010	Environmental management: comfort	6482	1.0
**5^th^ Functional Health Pattern:** Self-perception-self-concept						
**Nursing diagnoses**	**Code**	**Nursing outcomes**	**Code**	**Nursing interventions**	**Code**	**CVI**
Impaired verbal communication	00051	Communication	0902	Self-esteem enhancement	5400	0.80
**8^th^ Functional Health Pattern:** Coping-stress tolerance						
**Nursing diagnoses**	**Code**	**Nursing outcomes**	**Code**	**Nursing interventions**	**Code**	**CVI**
Fear	00148	Discomfort level	2109	Coping enhancement	5230	0.80
**9^th^ Functional Health Pattern:** Value-belief						
**Nursing diagnoses**	**Code**	**Nursing outcomes**	**Code**	**Nursing interventions**	**Code**	**CVI**
Spiritual distress	00066	Personal resiliency	1309	Spiritual growth facilitation	5426	0.80
**10^th^ Functional Health Pattern:** Activity-exercise						
**Nursing diagnoses**	**Code**	**Nursing outcomes**	**Code**	**Nursing interventions**	**Code**	**CVI**
Decreased activity tolerance	00298	Activity tolerance	0005	Exercise therapy: ambulation	0221	0.80
Risk for adult falls	00303	Knowledge: fall prevention	1828	Fall prevention	6490	1.0

To access all validated NNN linkage sets, use the following website: https://atenaeditora.com.br/index.php/catalogo/ebook/conjunto-de-ligacoes-nanda-i-noc-e-nic-para-emergencia-hospitalar-adulto-aplicacao-do-processo-de-enfermagem

## DISCUSSION

The use of SNT, such as NANDA-I, NOC and NIC, plays a crucial role in nursing practice standardization and organization, especially in highly complex environments, such as hospital emergencies^(11)^. Content validity by experts, as adopted in this study, is a fundamental step in ensuring the applicability of SNTs in specific contexts, allowing these tools to reflect the practical needs of care. Furthermore, validating linkages between NDs, NOs, and NIs strengthens clinical reasoning and evidence-based decision-making^(11)^.

In the hospital emergency room, the “Risk for impaired skin integrity” ND, “Tissue integrity: skin and mucous membranes” NO, and “Positioning” NI direct care for decubitus positions associated with support devices/pressure reducers and skin protectors. Its creation is justified by the prolonged stay during which patients may be subjected to observation to assess the clinical situation^(20,21)^ as a consequence of investigations into the presence of open lesions, originating from interventions in the postoperative period, increasing the potential for involvement by an infectious process^(20)^, loss or excess of heat triggered by involvement of the three layers of the skin, when in contact with the external environment^(21)^, as well as dehydration and decreased oxygenation, resulting from the aging process, in which dermis collagen and elastic fibers are degenerated^(22)^.

The linkage formed by the “Risk for ineffective thermoregulation” ND, “Thermoregulation” NO, and “Temperature regulation” NI is aimed at patients who require stabilization of body temperature and, consequently, reduction of energy expenditure, balance in gas transport, and minimization of impacts on the circulatory and respiratory systems, as well as hydroelectrolytic, hematologic, and hormonal alterations. Therefore, the suggested NAs are monitoring temperature, vital signs, skin temperature, and symptoms of hypothermia; using a warm blanket/heated blankets; administering warmed fluids; and monitoring skin color and temperature^(23-26)^.

Clinical practice focused on decreased cooperation, increased restlessness, changes in respiratory rate, altered arterial blood gases, and hypoxia guides the call directed by the “Impaired spontaneous ventilation” ND, which has been strengthened during the COVID-19 pandemic. For effective results, the “Respiratory status” NO and “Airway management” NI are recommended, directing nursing actions toward airway monitoring, ventilatory assistance, oxygen therapy, recording respiratory secretion characteristics, and medical record keeping^(25)^.

In this context, it is worth noting that three or more episodes of liquid or semi-liquid bowel movements per day, defined as diarrhea, is among the main reasons patients seek emergency care. Similar causal factors include cerebrovascular disorders, chronic patients using preventive laxatives through self-medication, which can lead to impaired motility, reduced reflexes, and an atonic colon, excess caloric intake, contamination through enteral feeding, gastrointestinal tract diseases, intolerances or allergies, and psychological factors. Therefore, given these conditions, it is suggested that the linkage between “Diarrhea” ND, “Bowel continence” NO, and “Diarrhea control” NI be investigated^(26)^.

Patients admitted to hospital emergency rooms tend to experience impaired comfort, characterized by a need for relief that transcends the physical, psychological, spiritual, and cultural dimensions. This characteristic is likely due to the environment and the degree of dependence that differs from the usual^(26)^. Therefore, this presentation guides the connection between “Impaired comfort” ND, “Comfort status: physical” NO, and “Environmental management: comfort” NI, leading nursing professionals to promote relief through NA as techniques of calm, tranquility, satisfaction and generally overcoming pain^(27,28)^.

The “Impaired verbal communication” ND is often directed at patients with decreased memory capacity, difficulty in transmitting and/or using a symbol system, those with stroke sequels, older adults, users at risk of falls, and patients with infections, incontinence, pressure injuries, dehydration, delirium, immobility, and depression^(29)^. Thus, the “Communication” NO requires attention to its verbal and nonverbal modes, as it is a basic human need that underpins all interpersonal relationships^(30)^. It is believed that its propagation in hospital emergencies encourages NAs such as bonding, transmission of security and calm, especially in palliative patients, as encouraging dialogue shows an improvement in self-esteem^(30)^.

The “Fear” ND portrays the feeling detected in the speech of companions of cancer patients, due to the fear of talking about the diagnosis due to its negative effects, such as suffering, worries, sadness, distress, exogenous intoxications, exacerbation of chronic processes and threatening sensations resulting from the COVID-19 pandemic process^(18)^. To mitigate this, the “Discomfort level” NO encourages emergency nursing professionals to the “Coping enhancement” NI, which guides NAs to promote a humanized environment. These include techniques to calm and improve coping, and explanations of terms and meanings that the family and patient are afraid of or do not understand, strengthening trust, bonding, and, consequently, improving health status^(28)^, which are palpable and sensitive in this context.

In hospital emergencies, admitted patients have, in their greatest proportion, an exponential risk of emotional and spiritual fragility, due to the risk of illness detected by hemodynamic destabilization or grief^(31,32)^, which impact disruptive and traumatic psychic processes. This subjectivity leads to the “Spiritual distress” ND, which provides an approach in the psychospiritual dimension, and to the “Resiliency” NO, in order to provide better coping with these demands^(31)^, when conciliated with NAs such as nurse-patient interaction, capable of providing understanding of suffering, refusals and adaptations, due to the openness to knowledge of the emotional aspect of the assisted person^(32)^ and overcoming taboos, which refer to the prohibition of a certain act due to superstitious belief and health.

The set of “Decreased activity tolerance” ND and “Activity tolerance” NO refers to individuals with insufficient endurance to complete required or desired daily activities, characterized by tiredness and fatigue. Its incidence is associated with the risk of functional decline, falls, hospitalization, institutionalization, and increased mortality. Cardiovascular and musculoskeletal system impairments are seen in older adults, those with respiratory symptoms, and those with acute chronic diseases such as congestive heart failure due to an imbalance between oxygen supply and demand. Generalized weakness manifested by signs and symptoms of decompensation resulting from systemic or pulmonary congestion^(6,12-26)^. To offer coping quality, the “Activity tolerance” NI is recommended, which leads to the “Locomotion” NA, applicable in emergencies^(12)^.

The set of “Risk for falls” ND and “Fall prevention behavior” NO highlights a potential adverse event, recurrent in hospital emergency room patients due to the high risk of worsening and the patient’s body being directed towards the ground. To prevent this deficit, NAs are adopted, such as care in locomotion and transfers, use of safety locks, and use of rails on stretchers and wheelchairs. These actions contribute to patient safety, minimizing injuries and sequels, reducing hospital stays, reducing costs, and reducing possible bleeding and fractures, with consequent legal liability for the healthcare team and the institution^(26,33)^.

These linkages allow recognizing the clinic of patients seeking clinical-surgical hospital emergencies, directing refined NNN linkages with feasible actions.

### Study limitations

The linkages proposed in cross-mapping, validated by experts, were numerous and extensive, making it impossible to provide a detailed list of the NAs applicable to each NI. Furthermore, although the study validated NNN linkages in terms of content, there was no practical application of these linkages in the clinical setting, which limits the assessment of their direct impact on the quality of care and patient outcomes. Future studies are recommended that apply these linkages to electronic medical records or nursing records to assess their effectiveness in care practice and their potential to optimize documentation and care delivery.

### Contributions to nursing, health, or public policy

The linkages structured by FHP theory enable the organization of nurses’ critical thinking regarding the health-disease process of individuals who, guided by anamnesis and physical examination, converge towards language standardization, evidencing targeted and satisfactory care, due to its synthesized format.

Thus, this technological innovation serves a dynamic and fast-paced scenario that has been incorporating its care methodologies, strengthening standardized communication, supported by the application of the intermediate NP stages (diagnosis, planning and nursing implementation), which provides the application of its construct in a computerized way in the emergency room of a multi-specialty hospital, which receives a high demand for clinical and surgical patients, adding to existing working conditions and valuing the service performed by the nursing class, meeting legislative demands.

## CONCLUSIONS

Twenty-nine NNN linkages were validated, consisting of 16 problem-focused NDs, 12 risk-focused NDs, and one syndrome-focused ND, in addition to 26 different NOs, 96 NIs, and 281 NAs, distributed across six FHPs, with an average CVI of 0.87. This validated model reinforces the systematization of clinical practice, enabling nurses to provide more precise, evidence-based, and results-oriented care. Standardizing NNN linkages can facilitate communication among healthcare professionals, improving continuity of care and patient safety, especially in highly complex settings such as hospital emergencies.

The study contributes not only to the validity of a theoretical-practical model, but also to the continuous improvement of nursing care, offering a structured guide that can be implemented in other hospital units, aiming to increase NP accuracy and efficiency.

## Data Availability

The research data are available within the article.
