# Supplementary material for: Standardized nursing terminology for adult emergency services: mapping and validity
Source: Rev Bras Enferm. 2026 Jan 26;79:e20240506. doi: 10.1590/0034-7167-2024-0506 (PMC12844539; doi:10.1590/0034-7167-2024-0506)
Supplement: Translation

# Translation: Terminologia padronizada em enfermagem para serviço hospitalar de emergência adulto: mapeamento e validação

**DOI:** 10.1590/0034-7167-2024-0506pt

**Authors:** Tatiane Félix Barbosa de Queiroz, Priscilla Alfradique de Souza, Natália Chantal Magalhães da Silva, Rosane Barreto Cardoso, Rodrigo Jensen, Daniella Fernandes de Almeida Santos

**Affiliations:** I Universidade Federal do Estado do Rio de Janeiro. Rio de Janeiro, Rio de Janeiro, Brasil; II Universidade Federal do Rio de Janeiro. Rio de Janeiro, Rio de Janeiro, Brasil; III Universidade de São Paulo. São Paulo, São Paulo, Brasil

**Keywords:** Terminologia Padronizada em Enfermagem, Processo de Enfermagem, Serviço Hospitalar de Emergência, Adulto, Estudo de Validação.

## Abstract

**Objetivos:**

mapear e validar ligações de terminologia padronizada de enfermagem aplicadas à emergência hospitalar adulto clínico-cirúrgica.

**Métodos:**

estudo metodológico realizado em unidade de emergência hospitalar adulto clínico-cirúrgica em Goiânia, Goiás, de julho de 2022 a janeiro de 2023. O estudo foi dividido em três etapas: coleta de sinais e sintomas em prontuários de pacientes; mapeamento cruzado por pesquisadoras e enfermeiros atuantes em emergência; e validação de conteúdo por especialistas.

**Resultados:**

foram validadas 29 ligações de terminologia, incluindo 16 diagnósticos de enfermagem com foco no problema, 12 diagnósticos de risco e um diagnóstico de síndrome, além de 26 resultados de enfermagem, 96 intervenções e 281 atividades. A média de concordância foi 0,87 entre os seis Padrões Funcionais de Saúde.

**Conclusões:**

as ligações validadas promovem a padronização dos diagnósticos, resultados e intervenções de enfermagem, aumentando a precisão e eficiência do processo de enfermagem nas emergências.

## INTRODUÇÃO

As unidades de urgência e emergência (UUE) hospitalar são portas de entrada para pacientes com condições agudas e risco iminente de morte. Concomitantemente, exige assistência qualificada aos indivíduos que buscam esses recintos. Portanto, os membros da equipe devem manter comunicação clara e conhecer os fluxos de trabalho para atuação dinâmica à singularidade de cada paciente, em virtude do cenário ser caracterizado pela alta rotatividade e complexidade que corrobora a postura de atuação célere e eficiente^(1,2)^.

Entre os profissionais atuantes neste cenário, os enfermeiros são desafiados a propiciar efetivo atendimento clínico através de assistência ágil e personalizada, baseada em conhecimentos sólidos, habilidades técnicas, e gestão eficaz do cuidado e da equipe, visando à rápida resolução das necessidades dos pacientes^(1)^. Dessa forma, diante dos recursos informatizados disponíveis neste meio, o prontuário eletrônico, acervo com informações de cada usuário, possui em sua estrutura possibilidade de implantação do Processo de Enfermagem (PE), método sistemático composto por cinco etapas cíclicas e interativas que orientam o registro e a execução da assistência prestada pela equipe de enfermagem^(3,4)^.

Estudos evidenciam que a aplicabilidade do PE atende às recomendações da Resolução n^o^ 736/2024 do Conselho Federal de Enfermagem, proporcionando: raciocínio clínico acurado e qualificação em suas ações; melhorias nos registros da enfermagem; organização, qualidade e segurança na assistência; otimização do tempo e do processo de trabalho; satisfação dos pacientes e profissionais; contribuições com benefícios para a instituição, pacientes e colaboradores^(4,5)^.

Para garantir a exequibilidade desse processo, recomenda-se o uso de terminologias padronizadas de enfermagem (TPE) e comunicação científica com emprego de classificações. Destacam-se a NANDA *International* (NANDA-I) para os diagnósticos de enfermagem (DE), a Classificação dos Resultados de Enfermagem (NOC), que mensura os resultados de enfermagem (RE), e a Classificação das Intervenções de Enfermagem (NIC), que organiza as intervenções de enfermagem (IE)^(6-8)^, que integradas são intituladas ligação NNN. Consecutivamente, são potencializas quando as instituições de saúde fundamentam suas práticas na filosofia institucional, oferecendo um atendimento alinhado às necessidades dos pacientes^(6-11)^.

Estudos apontam que as TPE melhoram a comunicação entre os profissionais de saúde e otimiza a tomada de decisões, especialmente em ambientes de alta complexidade^(11)^. Registros construídos com essas terminologias para meios informatizados de UUE hospitalar são considerados instrumentos tecnológicos funcionais para organizar, gerir, documentar e evidenciar os cuidados e assistência da equipe de enfermagem^(12)^. Além disso, a estruturação dessas terminologias alinhadas com modelos teóricos potencializa a sistematização do cuidado, contribuindo para uma assistência mais direcionada, eficaz e segura^(9,10)^.

Entre os modelos teóricos que orientam a coleta de dados e a definição do plano de cuidados, destacam-se os Padrões Funcionais de Saúde (PFS), desenvolvidos por Marjory Gordon em 1982. Essa tipologia é estruturada em 11 categorias, incluindo: percepção e controle de saúde; nutricional-metabólico; eliminações; cognitivo-perceptivo; autopercepção e autoconceito; desempenho de papéis e relacionamentos; sexual-reprodutivo; resposta e tolerância ao estresse; crenças e valores; atividade e exercício; e sono e repouso^(9,10)^.

Na literatura, a validação de instrumentos para registro do PE construídos com TPE norteada por modelo teórico proporciona descrição da documentação da prática do enfermeiro emergencista, com respaldo legal, técnico, ético e científico^(13)^.

Embora estudos revelem as contribuições das TPE para a assistência à saúde, principalmente na emergência hospitalar, que atende recorrentemente pacientes instáveis e com elevado risco de deterioramento, os estudos que elencam terminologias de enfermagem completas para esse cenário são incipientes, caracterizando este estudo como pertinente diante do elucidado.

## OBJETIVOS

Mapear e validar ligações de Terminologia Padronizada em Enfermagem aplicadas à emergência hospitalar adulto clínico-cirúrgica.

## MÉTODOS

### Aspectos éticos

Estudo aprovado pelos Comitês de Ética em Pesquisa da Universidade Federal do Rio de Janeiro, como instituição proponente, e da Universidade Federal de Goiás, como instituição coparticipante.

### Desenho, período e local do estudo

Trata-se de estudo metodológico, em que foi utilizado o instrumento *Strengthening the reporting of observational studies in epidemiology* ^(14)^ como norte metodológico. O estudo foi desenvolvido em três etapas, no período de julho de 2022 a janeiro de 2023, em uma emergência hospitalar adulto clínico-cirúrgica de uma instituição privada de alta complexidade localizada em Goiânia, Goiás.

A primeira etapa, realizada em julho de 2022, consistiu na coleta de sinais e sintomas que motivaram a procura dos pacientes por atendimento na emergência. A segunda etapa, realizada de agosto a novembro de 2022, envolveu a construção do mapeamento cruzado^(15)^, elaborado por enfermeiros atuantes na unidade de emergência e pesquisadoras. A terceira etapa transcorreu de dezembro de 2022 a janeiro de 2023, sendo realizada a validação de conteúdo por especialistas.

### População ou amostra; critérios de inclusão e exclusão

Os prontuários foram selecionados por amostragem não intencional. Foram incluídos 900 prontuários que continham sinais e sintomas registrados no ato da consulta de enfermagem, de atendimentos realizados de janeiro de 2019 a dezembro de 2021, no período diurno e noturno.

Quanto aos enfermeiros com prática assistencial em UUE, foram convidados 15, com participação de nove. Os critérios de inclusão foram possuir atuação no setor de emergência por regime de lotação ou cobertura de plantão ≥ 12 horas. Os critérios de exclusão foram ser enfermeiros com COVID-19 no período da pesquisa.

Foram selecionados por amostragem não probabilística intencional e método *snowball* ^(16)^ 47 possíveis especialistas. Os critérios de inclusão basearam-se na adaptação dos critérios de Fehring^(17)^, exigindo uma pontuação mínima de 5 pontos. A pontuação foi distribuída da seguinte forma: doutorado em enfermagem (4 pontos); mestrado em enfermagem (3 pontos); titulação de doutor e/ou mestre com estudos voltados a DEs, teoria de enfermagem, PE ou TPE (2 pontos); especialização em urgência e/ou emergência (2 pontos); experiência assistencial em urgência e/ou emergência por um período igual ou superior a 1 ano (1 ponto); publicação de artigos sobre PE, PFS, TPE ou urgência/emergência (2 pontos); e participação em eventos científicos nos últimos dois anos relacionados ao tema do estudo (1 ponto). Foram excluídos os especialistas que preencheram o formulário de maneira incompleta.

### Protocolo do estudo

Os sinais e sintomas extraídos dos prontuários foram coletados manualmente por uma das pesquisadoras e inseridos em uma planilha do *Microsoft Excel*, onde foram organizados por PFS e quantificado.

Posteriormente, os enfermeiros com prática assistencial na UUE receberam um *link* do *Google Forms* composto por três seções: carta convite, Termo de Consentimento Livre e Esclarecido (TCLE) e questionário de caracterização (idade, sexo, tempo de profissão, renda mensal e especialização). Após a coleta dessas informações, foram realizadas videoconferências entre os enfermeiros com prática assistencial em UUE e as pesquisadoras, para construção do mapeamento cruzado^(15)^.

No formulário, cada seção correspondia a um PFS, contendo o título com definição, sinais e sintomas, ligações NNN, correspondentes às classificações NANDA-I 2021-2023^(6)^, NOC 2022^(7)^ e NIC 2022^(8)^, além de um espaço para sugestões e comentários. Essa etapa teve duração de 20 dias consecutivos. Posteriormente, foi realizada a validação de conteúdo do mapeamento cruzado por especialistas.

Os especialistas receberam um formulário *Google Forms* composto por quatro seções: carta convite; TCLE; questionário de caracterização (sexo, idade, local de residência, informações profissionais); e mapeamento cruzado para validação do conteúdo das ligações NNN. As ligações foram validadas em três eixos (1) clareza; 2) objetividade; e 3) importância), utilizando uma escala Likert composta por cinco opções (A - concordo totalmente (1,0); B - concordo (0,75); C - parcialmente (0,5); D - discordo (0,25); E - discordo totalmente (0,0)^(16)^, sendo possível a seleção de uma opção em cada eixo. No final de cada seção, havia um espaço para comentários e sugestões.

### Análise dos resultados e estatística

Os especialistas julgaram o grau de concordância e confiabilidade das ligações NNN através do Índice de Validade de Conteúdo (IVC)^(16,17)^ por conjunto, PFS e na íntegra. O valor de referência para confiabilidade e excelência adotado foi ≥ 0,80^(16,17)^. Para o cálculo do IVC de cada item, foi considerado o número total de opções (5) atribuído pelos especialistas, dividido pelo número total de respostas.

Utilizou-se o *software Microsoft Excel* para tabulação dos dados com posterior análise descritiva. Para a análise, empregou-se a estatística descritiva, que consiste em um meio de organizar e resumir as características principais observadas em um conjunto de dados, permitindo ao pesquisador melhor compreensão dos dados estudados^(16)^.

Ressalta-se que a nova edição da NANDA-I 2024-2026^(18)^ apresenta revisão de todos os DE validados. Entre eles, 17 apresentam modificações na definição, edição, adições e/ou remoções de características definidoras, fatores de risco e fatores relacionados, bem como substituição de 12 DE, sendo cinco de risco e sete com foco no problema. Quanto à versão mais atual da NOC 2024^(19)^, não houve modificações nos RE validados.

## RESULTADOS

Foram identificados 2.267 sintomas que, após serem distribuídos em oito PFS orientaram o mapeamento cruzado das 76 ligações NNN com 64 diferentes DE, 59 RE e 122 grupos de IE que acarretaram no levantamento de 602 atividades de enfermagem (AE). O Quadro 1 apresenta um recorte das ligações NNN estabelecidas.

**Quadro 1:** Padrões Funcionais de Saúde, sinais e sintomas com ocorrências, e principais terminologias da NANDA-*International*, Classificação dos Resultados de Enfermagem e Classificação das Intervenções de Enfermagem elencadas para emergência hospitalar adulto clínico-cirúrgico, Goiânia, Goiás, Brasil, 2023

2º Padrão Funcional de Saúde: Nutricional-metabólico				
***Sinais e sintomas agrupados com ocorrência:*** Alterações gastrointestinais, alterações cardíacas, alterações sistêmicas, alterações pressóricas, desconfortos, febre, alterações sistêmicas, sinais flogísticos (n=1.189)				
**Diagnósticos de enfermagem**		**Resultados de enfermagem**		**Intervenções de enfermagem**
Risco de termorregulação ineficaz		Termorregulação		Regulação da temperatura
Ventilação espontânea prejudicada		Estado respiratório: troca gasosa		Monitorização respiratória
**3º Padrão Funcional de Saúde:** Eliminações				
***Sinais e sintomas agrupados com ocorrência:*** Diarreia, diurese, incontinência urinária (n=367)				
**Diagnósticos de enfermagem**		**Resultados de enfermagem**		**Intervenções de enfermagem**
Retenção urinária		Eliminação urinária		Cuidados na retenção urinária
Diarreia		Continência intestinal		Controle da diarreia
**4º Padrão Funcional de Saúde:** Cognitivo-perceptivo				
***Sinais e sintomas agrupados com ocorrência:*** Traumas, quedas, algias, confusão, rebaixamento nível de consciência (n=24)				
**Diagnósticos de enfermagem**		**Resultados de enfermagem**		**Intervenções de enfermagem**
Conforto prejudicado		Estado de conforto: físico		Controle do ambiente: conforto
Dor aguda		Estado de conforto		Controle de dor aguda
**5º Padrão Funcional de Saúde:** Autopercepção e autoconceito				
***Sinais e sintomas agrupados com ocorrência:*** Alterações na fala (n=20)				
**Diagnósticos de enfermagem**		**Resultados de enfermagem**		**Intervenções de enfermagem**
Comunicação verbal prejudicada		Comunicação		Fortalecimento da autoestima
**8º Padrão Funcional de Saúde:** Resposta e tolerância ao estresse				
***Sinais e sintomas agrupados com ocorrência:*** Estresse, euforia, tristeza, ansiedade, sobrecarga de trabalho (n=211)				
**Diagnósticos de enfermagem**		**Resultados de enfermagem**		**Intervenções de enfermagem**
Medo		Nível de desconforto		Melhora do enfrentamento
**9º Padrão Funcional de Saúde:** Crença e valor				
***Sinais e sintomas agrupados com ocorrência:*** Sensações e sentimentos (n=20)				
**Diagnósticos de enfermagem**		**Resultados de enfermagem**		**Intervenções de enfermagem**
Angústia espiritual		Resiliência pessoal		Promoção da resiliência
**10º Padrão Funcional de Saúde:** Atividade e exercício				
***Sinais e sintomas agrupados com ocorrência:*** Distúrbios motores e cansaço (n=20)				
**Diagnósticos de enfermagem**		**Resultados de enfermagem**		**Intervenções de enfermagem**
Mobilidade física prejudicada		Mobilidade		Assistência no autocuidado: transferências
**11º Padrão Funcional de Saúde:** Sono e repouso				
***Sinais e sintomas agrupados com ocorrência:*** Insônia (n=13)				
Insônia		Sono		Melhora do sono

Em relação à representação do PFS, o segundo (nutricional-metabólico) apresentou 1.189 sinais e sintomas, seguido pelo terceiro (eliminações), com 367, e o oitavo (resposta e tolerância ao estresse), com 211. Em contrapartida, o décimo primeiro (sono e repouso) foi o menos recorrente, com 13 termos.

Quanto aos nove enfermeiros com prática assistencial em UUE participantes da segunda etapa do estudo, a faixa etária predominante foi de 25 a 40 anos (44,5%), com maioria do sexo feminino (67%), 1 a 5 anos de profissão (55,5%), renda mensal entre um e três salários mínimos, e especializados em urgência, emergência ou Unidade de Terapia Intensiva (44,5%).

Em relação aos especialistas que participaram da etapa de validação de conteúdo, a faixa etária predominante foi entre 30 e 35 anos, com três especialistas (60%). A maioria era do sexo masculino (60%), e quatro especialistas (80%) residiam no Brasil, enquanto um (20%) era da Colômbia. No que diz respeito ao vínculo profissional, a maior parte tinha maior tempo de atuação na docência (60%) e também possuía experiência prática no setor de emergência (60%), embora nenhum deles tivesse concluído uma especialização *lato sensu* em UUE. A pontuação nos critérios de inclusão variou entre 9 e 15, com uma média de 11,6.

Durante a validação de conteúdo pelos especialistas, foi sugerida a substituição de alguns RE e a adição de AEs, resultando modificações em três PFS: nutricional-metabólico; eliminações; e perceptivo-cognitivo. No total, foram validadas 29 ligações NNN, compostas por 16 DE com foco no problema, 12 de risco e uma de síndrome, além de 26 diferentes RE e 96 IE. As ligações foram distribuídas entre seis PFS: 2º - nutricional-metabólico (0,87); 3º - eliminações (0,84); 4º - perceptivo-cognitivo (1,0); 8º - resposta e tolerância ao estresse (0,90); 9º - crença e valor (0,80); e 10º - atividade e exercício (0,90). As ligações validadas foram listadas com códigos das respectivas terminologias, visando facilitar a implantação na prática, através do prontuário eletrônico do paciente, utilizando *softwares*, como o disposto na emergência local (Quadro 2).

**Quadro 2:** Padrões Funcionais de Saúde com principais ligações NANDA-*International*, Classificação dos Resultados de Enfermagem e Classificação das Intervenções de Enfermagem validados pelos especialistas, Goiânia, Goiás, Brasil, 2023

2º Padrão Funcional de Saúde: Nutricional-metabólico						
**Diagnósticos de Enfermagem**	**Código**	**Resultados de Enfermagem**	**Código**	**Intervenções de Enfermagem**	**Código**	**IVC**
Risco de integridade da pele prejudicada	00047	Integridade tissular: pele e mucosas	1101	Posicionamento	0840	0,93
Risco de termorregulação ineficaz	00274	Termorregulação	0800	Regulação da temperatura	3900	0,86
Troca de gases prejudicada	00033	Estado respiratório	0415	Controle de vias aéreas	3140	1,0
**3º Padrão Funcional de Saúde:** Eliminações						
**Diagnósticos de Enfermagem**	**Código**	**Resultados de Enfermagem**	**Código**	**Intervenções de Enfermagem**	**Código**	**IVC**
Diarreia	00013	Continência intestinal	0500	Controle da diarreia	0460	0,80
**4º Padrão Funcional de Saúde:** Cognitivo-perceptivo						
**Diagnósticos de Enfermagem**	**Código**	**Resultados de Enfermagem**	**Código**	**Intervenções de Enfermagem**	**Código**	**IVC**
Conforto prejudicado	00214	Estado de conforto: físico	2010	Controle do ambiente: conforto	6482	1,0
**5º Padrão Funcional de Saúde:** Autopercepção e autoconceito						
**Diagnósticos de Enfermagem**	**Código**	**Resultados de Enfermagem**	**Código**	**Intervenções de Enfermagem**	**Código**	**IVC**
Comunicação verbal prejudicada	00051	Comunicação	0902	Fortalecimento da autoestima	5400	0,80
**8º Padrão Funcional de Saúde:** Resposta e tolerância ao estresse						
**Diagnósticos de Enfermagem**	**Código**	**Resultados de Enfermagem**	**Código**	**Intervenções de Enfermagem**	**Código**	**IVC**
Medo	00148	Nível de desconforto	2109	Melhora do enfrentamento	5230	0,80
**9º Padrão Funcional de Saúde:** Crença e valor						
**Diagnósticos de Enfermagem**	**Código**	**Resultados de Enfermagem**	**Código**	**Intervenções de Enfermagem**	**Código**	**IVC**
Angústia espiritual	00066	Resiliência pessoal	1309	Facilitação do crescimento espiritual	5426	0,80
**10º Padrão Funcional de Saúde:** Atividade e exercício						
**Diagnósticos de Enfermagem**	**Código**	**Resultados de Enfermagem**	**Código**	**Intervenções de Enfermagem**	**Código**	**IVC**
Tolerância de atividade diminuída	00298	Tolerância à atividade	0005	Terapia com exercício: deambulação	0221	0,80
Risco de queda em adultos	00303	Conhecimento: prevenção de quedas	1828	Prevenção contra quedas	6490	1,0

Para acesso a todos os conjuntos de ligações NNN validados, utilizar o seguinte site: https://atenaeditora.com.br/index.php/catalogo/ebook/conjunto-de-ligacoes-nanda-i-noc-e-nic-para-emergencia-hospitalar-adulto-aplicacao-do-processo-de-enfermagem

## DISCUSSÃO

A utilização de TPE, como NANDA-I, NOC e NIC, desempenha um papel crucial na padronização e organização da prática de enfermagem, especialmente em ambientes de alta complexidade, como as emergências hospitalares^(11)^. A validação de conteúdo por especialistas, como adotada neste estudo, é uma etapa fundamental para garantir a aplicabilidade das TPE em contextos específicos, permitindo que essas ferramentas reflitam as necessidades práticas da assistência. Além disso, a validação de ligações entre DE, RE e IE fortalece o raciocínio clínico e a tomada de decisão baseada em evidências^(11)^.

No setor de emergência hospitalar, a ligação composta pelo DE “Risco de integridade da pele prejudicada”, RE “Integridade tissular: pele e mucosas” e IE “Posicionamento” direciona a cuidados com decúbitos associado a dispositivos de apoio/redutores de pressão e protetores cutâneos. Seu levantamento é justificado pela estadia prolongada em que o paciente poderá ser submetido à observação para avaliação da situação clínica^(20,21)^ como consequência de investigações da presença de lesões abertas, oriundas de intervenções em período pós-operatório, elevando o potencial de acometimento por processo infeccioso^(20)^, perda ou excesso de calor desencadeado pelo acometimento das três camadas da pele, quando em contato com o ambiente externo^(21)^, bem como desidratação e diminuição da oxigenação, decorrentes do processo de envelhecimento, no qual as fibras colágenas e elásticas da derme são degeneradas^(22)^.

A ligação formada pelo DE “Risco de termorregulação ineficaz”, RE “Termorregulação” e IE “Regulação da temperatura” é direcionada para pacientes que necessitam de estabilização da temperatura corporal e, consequentemente, redução do gasto energético, equilíbrio no transporte de gases e minimização de impactos nos sistemas circulatório, respiratório, alterações hidroeletrolíticas, hematológicas e hormonais. Assim, as AE sugeridas são monitorar a temperatura, sinais vitais, temperatura da pele e sintomas de hipotermia; utilizar manta quente/cobertores aquecidos; administrar fluídos aquecidos; e monitorar cor e temperatura da pele^(23-26)^.

A clínica pautada em cooperação diminuída, inquietação aumentada, alterações na frequência respiratória, gases sanguíneos arteriais alterados e hipóxia orienta a ligação direcionada pelo DE “Troca de gases prejudicada”, potencializado no período pandêmico da COVID-19. Para efetivos resultados, sugerem-se o RE “Estado respiratório” e IE “Controle de vias aéreas”, que direcionam as ações de enfermagem a cuidados com a monitorização das vias aéreas, assistência ventilatória, fornecimento de oxigenoterapia, anotações das características das secreções respiratórias e registros em prontuário^(25)^.

Nessa sequência, ressalta-se que a presença de três ou mais episódios de evacuações líquidas ou semilíquidas/dia, definida como diarreia, está entre os principais motivos de busca dos pacientes nas emergências. Entre os motivos causais semelhantes, estão transtorno cerebrovascular, doentes crônicos em uso de laxativos preventivos por automedicação, ocasionando prejuízos na motilidade, redução de reflexos e cólon atônico, aporte calórico em excesso, contaminação através do manuseio da dieta enteral, doenças do trato gastrointestinal, intolerâncias ou alergias, e fatores psicológicos. Portanto, diante desses quadros, sugere-se o levantamento da ligação entre DE “Diarreia”, RE “Continência intestinal” e IE “Controle de diarreia”^(26)^.

Pacientes admitidos nas emergências hospitalares tendem a apresentar conforto prejudicado, compreendido pela necessidade de alívio que transcende o físico, psíquico, espiritual e cultural. Essa característica é propensa ao ambiente e ao grau de dependência que difere do habitual^(26)^. Portanto, essa apresentação orienta a ligação entre DE “Conforto prejudicado”, RE “Estado de conforto: físico” e IE “Controle do ambiente: conforto”, conduzindo profissionais de enfermagem à promoção de alívio por meio de AE como técnicas de calma, tranquilidade, satisfação e geralmente superação da dor^(27,28)^.

O DE “Comunicação verbal prejudicada” é direcionado frequentemente a pacientes com capacidade diminuída na memória, dificuldade em transmitir e/ou usar um sistema de símbolos, portadores de sequela de acidente vascular encefálico, idosos, usuários com risco de quedas, e pacientes com infecções, incontinência, lesões por pressão, desidratação, delírio, imobilidade e depressão^(29)^. Assim, o RE “Comunicação” exige atenção aos seus modos verbal e não verbal, por se tratar de uma necessidade humana básica que fundamenta todas as relações interpessoais^(30)^. Acredita-se que sua propagação na emergência hospitalar incentiva AEs como vínculo, transmissão de segurança e calma, principalmente em pacientes paliativos, pois o estímulo ao diálogo evidencia melhora na autoestima^(30)^.

O DE “Medo” retrata o sentimento detectado na fala de acompanhantes de pacientes oncológicos, devido ao receio de falar sobre o diagnóstico devido aos seus efeitos negativos, como sofrimento, preocupações, tristeza, aflição, intoxicações exógenas, agudização de processos crônicos e sensações ameaçadoras resultantes do processo pandêmico da COVID-19^(18)^. Para mitigá-lo, o RE “Nível de desconforto” impulsiona os profissionais de enfermagem da emergência à IE “Melhora do enfrentamento”, que orienta AEs que promovam ambiente humanizado. Entre elas, estão a técnica para acalmar e melhorar o enfrentamento, e explicação de termos e significados que a família e o paciente têm receio ou não compreendem, fortalecendo confiança, vínculo e, consecutivamente, melhora do estado de saúde^(28)^, que são palpáveis e sensíveis neste contexto.

Nas emergências hospitalares, os pacientes admitidos possuem, em sua maior proporção, risco exponencial de fragilidade emocional e espiritual, devido ao risco de adoecimento detectado por desestabilização hemodinâmica ou luto^(31,32)^, que impactam processos disruptivos e traumáticos psíquicos. Essa subjetividade direciona ao DE “Angústia espiritual”, que proporciona abordagem na dimensão psicoespiritual, e ao RE “Resiliência”, a fim de proporcionar melhor enfrentamento a essas demandas^(31)^, quando conciliadas a AEs como interação enfermeiro e paciente, capaz de proporcionar compreensão do sofrimento, recusas e adaptações, devido à abertura ao conhecimento do aspecto emocional do assistido^(32)^ e superação a tabus, que referem à proibição de determinado ato por crença supersticiosa e saúde.

O conjunto composto por DE “Tolerância à atividade diminuída” e RE “Tolerância à atividade” se refere a indivíduos com resistência insuficiente para completar as atividades diárias exigidas ou desejadas, caracterizadas pelo cansaço e fadiga. Sua incidência está associada ao risco de declínio funcional, quedas, hospitalização, institucionalização e aumento da mortalidade, acometimentos no sistema cardiovascular e musculoesquelético percebidos em idosos, sintomáticos respiratórios e portadores de doenças crônicas agudizadas como a Insuficiência Cardíaca Congestiva, devido ao desequilíbrio entre oferta e demanda de oxigênio, fraqueza generalizada traduzida por sinais e sintomas da descompensação, oriundos da congestão sistêmica ou pulmonar^(6,12,26)^. Para ofertar qualidade de enfrentamento, orienta-se a IE “Tolerância à atividade”, que conduz a AE “Locomoção”, aplicável na emergência^(12)^.

O conjunto norteado pelo DE “Risco de quedas” e RE “Comportamento de prevenção de queda” evidencia um potencial evento adverso, recorrente nos pacientes das emergências hospitalares devido ao alto risco de agravamento e direcionamento do corpo do paciente contra o solo. Para prevenir esse déficit, adotam-se AE como cuidados na locomoção e na transferência, uso de travas de segurança, e uso de grades em macas e cadeira de rodas, que corroboram a segurança do paciente, minimizando ferimentos, sequelas, redução do tempo de internação, diminuição de custos, possíveis hemorragias e fraturas com consequente responsabilização legal da equipe de saúde e da instituição^(26,33)^.

Essas ligações permitem o reconhecimento da clínica dos pacientes que buscam as emergências hospitalares clínico-cirúrgicas, direcionando as ligações NNN refinadas com ações exequíveis.

### Limitações do estudo

As ligações propostas no mapeamento cruzado, validadas pelos especialistas, foram numerosas e extensas, o que impossibilitou a listagem detalhada das AE aplicáveis a cada IE. Além disso, embora o estudo tenha validado as ligações NNN em termos de conteúdo, não houve uma aplicação prática dessas ligações no ambiente clínico, o que limita a avaliação do impacto direto na qualidade da assistência e nos desfechos dos pacientes. Recomenda-se a realização de estudos futuros que apliquem essas ligações em prontuários eletrônicos ou registros de enfermagem, a fim de avaliar sua efetividade na prática assistencial e seu potencial para otimizar a documentação e o cuidado prestado.

### Contribuições para a área da enfermagem, saúde ou política pública

As ligações estruturadas pela teoria dos PFS possibilitam a organização do pensamento crítico do enfermeiro diante do processo de saúde-doença dos indivíduos que, direcionados pela anamnese e exame físico, convergem para padronização de linguagem, evidenciando assistência direcionada e satisfatória, devido ao seu formato sintetizado.

Assim, esta inovação tecnológica atende um cenário dinâmico e célere que vem incorporando suas metodologias assistenciais, fortalecendo a comunicação padronizada, sustentada pela aplicação das etapas intermediárias (diagnóstico, planejamento e implementação de enfermagem) do PE, que proporciona a aplicação do seu construto de forma informatizada na emergência de um hospital de múltiplas especialidades, que recebe acentuada demanda de pacientes clínicos e cirúrgicos, agregando nas condições de trabalho existentes e valorizando o serviço executado pela classe de enfermagem, atendimento às demandas legislativas.

## CONCLUSÕES

Foram validadas 29 ligações NNN, formadas de 16 DE com foco no problema, 12 de risco e um de síndrome, além de 26 diferentes RE, 96 IE e 281 AE, distribuídos em seis PFS, com IVC médio de 0,87. Esse modelo validado reforça a sistematização da prática clínica, permitindo que os enfermeiros forneçam um cuidado mais preciso, baseado em evidências e orientado para resultados. A padronização das ligações NNN poderá facilita a comunicação entre os profissionais de saúde, aprimorando a continuidade do cuidado e a segurança do paciente, especialmente em contextos de alta complexidade, como as emergências hospitalares.

O estudo contribui não apenas para a validação de um modelo teórico-prático, mas também para a melhoria contínua da assistência de enfermagem, oferecendo um guia estruturado que pode ser implementado em outras unidades hospitalares, visando aumentar a acurácia e eficiência do PE.

## Data Availability

Os dados da pesquisa estão disponíveis no corpo do artigo.
